# Whole genome sequencing analysis of* Mycobacterium tuberculosis* reveals circulating strain types and drug-resistance mutations in the Philippines

**DOI:** 10.1038/s41598-024-70471-x

**Published:** 2024-08-23

**Authors:** Linfeng Wang, Dodge R. Lim, Jody E. Phelan, Lorenzo T. Reyes, Alma G. Palparan, Maria Guileane C. Sanchez, Louella Abigail A. Asuncion, Ma. Angelica A. Tujan, Inez Andrea P. Medado, Chona Mae A. Daga, Jo-Hannah S. Llames, Satoshi Mitarai, Yoshiro Murase, Yuta Morishige, Concepcion F. Ang, John Carlo M. Malabad, Jaime C. Montoya, Julius C. Hafalla, Susana Campino, Martin L. Hibberd, Cecilia G. Ama, Ramon P. Basilio, Eva Maria Cutiongoco-De La Paz, Taane G. Clark

**Affiliations:** 1 Department of Infection Biology, Faculty of Infectious and Tropical Diseases, London School of Hygiene & Tropical Medicine, London, WC1E 7HT UK; 2 National Tuberculosis Reference Laboratory, Research Institute for Tropical Medicine, Department of Health, Filinvest, Alabang, Muntinlupa City, Philippines; 3 Molecular Biology Laboratory, Research Institute for Tropical Medicine, Department of Health, Filinvest, Alabang, Muntinlupa City, Philippines; 4 Department of Epidemiology and Biostatistics, Research Institute for Tropical Medicine, Department of Health, Filinvest, Alabang, Muntinlupa City, Philippines; 5 Philippine Genome Center, University of Philippines Diliman, Quezon City, Philippines; 6 Department of Mycobacterium Reference and Research, Research Institute of Tuberculosis, Japan Anti-Tuberculosis Association, Tokyo, Japan; 7 College of Medicine, University of the Philippines, Manila, Philippines; 8 Department of Science and Technology, DOST Compound, Bicutan, Taguig City, Philippine, Philippines; 9 Department of Infectious Disease Epidemiology, Faculty of Epidemiology and Population Health, London School of Hygiene and Tropical Medicine, London, WC1E 7HT UK

**Keywords:** Epidemiology, Population screening, Tuberculosis, Genomics

## Abstract

The Philippines is a high-incidence country for tuberculosis, with the increasing prevalence of multi- (MDR-TB) and extensively-drug (XDR-TB) resistant *Mycobacterium tuberculosis* strains posing difficulties to disease control. Understanding the genetic diversity of circulating strains can provide insights into underlying drug resistance mutations and transmission dynamics, thereby assisting the design of diagnostic tools, including those using next generation sequencing (NGS) platforms. By analysing genome sequencing data of 732 isolates from Philippines drug-resistance survey collections spanning from 2011 to 2019, we found that the majority belonged to lineages L1 (531/732; 72.5%) and L4 (European-American; n = 174; 23.8%), with the Manila strain (L1.2.1.2.1) being the most prominent (475/531). Approximately two-thirds of isolates were found to be at least MDR-TB (483/732; 66.0%), and potential XDR-TB genotypic resistance was observed (3/732; 0.4%), highlighting an emerging problem in the country. Genotypic resistance was highly concordant with laboratory drug susceptibility testing. By finding isolates with (near-)identical genomic variation, five major clusters containing a total of 114 isolates were identified: all containing either L1 or L4 isolates with at least MDR-TB resistance and spanning multiple years of collection. Closer inspection of clusters revealed transmission in prisons, some involving isolates with XDR-TB, and mutations linked to third-line drug bedaquiline. We have also identified previously unreported mutations linked to resistance for isoniazid, rifampicin, ethambutol, and fluoroquinolones. Overall, this study provides important insights into the genetic diversity, transmission and circulating drug resistance mutations of *M. tuberculosis* in the Philippines, thereby informing clinical and surveillance decision-making, which is increasingly using NGS platforms.

## Introduction

Tuberculosis (TB), caused by *Mycobacterium tuberculosis*, is a prevalent infectious disease in the Philippines. With over 741 K cases and 61 K deaths in 2021 alone^1^, the Philippines is the country with the second highest active disease burden, after China^1^. Approximately 70 Filipinos die daily from TB. Worryingly, increasing HIV prevalence and a high burden of multi-drug resistance (MDR-TB) to isoniazid (INH) and rifampicin (RIF) treatments pose serious challenges for effective control^3^. These challenges are exacerbated by a serious gap between those expected to have MDR-TB (2% new, 21% re-treatment cases) and those detected and subsequently put on treatment, with detrimental consequences including poorer treatment outcomes and strains progressing to extensively drug-resistance (XDR-TB)^2^. Novel applications of whole genome (WGS) and targeted (candidate) amplicon sequencing (Amp-seq) using next generation (NGS) technologies can provide insights into the underlying drug resistance mutations and profiles for clinical and surveillance decision-making. However, there is a lack of WGS studies of *M. tuberculosis* from low- and middle-income countries, such as the Philippines, where the prevalence and burden of TB tend to be greatest.

In the Philippines, genomic data for local *M. tuberculosis* isolates is scarce, but previous work has shown that ‘ancient’ (lineage L1) and ‘modern’ lineages (L2 and L4) are present in the country^3^. Members of the EA12-Manila clade (L1.2.1.2.1) are known to be highly associated with the Filipino population^3^, with a molecular barcode established to rapidly identify this strain type^4^. Here, we present the results of sequencing 732 isolates recently collected between 2011 and 2019, including from prison populations. We analysed the temporal evolution of drug resistance and clustering by geography and found evidence of the transmission of L4 and MDR-TB strains across the islands. More generally, TB transmission in prison poses a challenge to infection control, with the potential spread of infections within and outside the institution, and several studies have used sequencing to understand links between isolates^5–8^, and inform a public health response. Within confined correctional spaces, TB thrives, driven by overcrowding and potentially limited ventilation and healthcare access, thereby amplifying the risk of transmission. The flow of individuals into and out of prisons poses a broader public health concern, and the response necessitates targeted screening, rapid diagnosis, and tailored treatments.

Mixed infections in TB introduce complex genetic interactions that impact disease severity, treatment response, and transmission dynamics. The co-existence of diverse clones within a host complicates treatment regimes, and genetic investigation of the bacterial population can inform patient management^9^. Platforms such as Illumina and the development of portable devices, such as Oxford Nanopore Technology, have ushered in a transformative era, making WGS and Amp-seq a cornerstone in deciphering the genome variation and diversity of *M. tuberculosis*. WGS is now widely used to identify drug resistance markers to guide treatment, determine phylogenetic relatedness and potential transmission events, inform surveillance and infection control decision-making, and discover new targets for drugs and vaccines. Applying such technologies and assays will have the greatest impact in settings with high TB burden, such as The Philippines. In this study, we utilised WGS data from a convenience dataset to explore the dynamics of drug resistance development, transmission, and the complexities of mixed infections in the Philippines. By conducting an integrated analysis of WGS data, we aimed to establish a baseline characterisation of genomic diversity within the country. This foundational insight will inform future routine applications of NGS, guiding public health decision-making and helping to reduce the high burden of TB.

## Results

### Study population

The study analysed a convenience sample of 732 M*. tuberculosis* isolates, all with sequencing coverage more than 25-fold (range: 27- to 2191-fold) and collected from 2011 to 2019 across three studies (see “Materials and methods”). They were collected from 671 TB patients, but eight of the isolates were found to be sourced from mixed infections and subsequently excluded from further population genomic analysis (Table S1). The study patients (n = 671) were predominantly male (70.5%), with a median age of 42 (range 14–83) years, mostly from Luzon Island (74.4%), and 18 (2.7%) contributed more than one isolate at different time points (range: 2 to 14 isolates) (Table 1). The 724 isolates belong predominantly to lineage 1 (L1; 531, 73.3%), with the Manila strain (lineage 1.2.1.2.1) being the most common (89.5%; 475/531) and were found in all three major islands (Luzon, Visayas, and Mindanao) (Table 2). Lineage 4 (L4) was the second most common lineage (24.0%, n = 174), followed by lineage 2 (L2; 2.5%; n = 18) and lineage 3 (L3; 0.1%, n = 1). To avoid bias in some population-based analyses, we used a single representative isolate for each patient (n = 671), whereas for the 18 patients with multiple isolates, we used the most recent sample.

**Table 1:** *Mycobacterium tuberculosis* individual (N = 671).

Characteristic	N (median)	% (range)
Age (years)	42	(14-83)
Gender		
Male	473	70.5
Female	149	22.2
Unknown	49	7.3
Location*		
National Capital Region, Luzon	200	29.8
Calabarzon, Luzon	125	18.6
Other Luzon	174	25.9
Mindanao	113	16.8
Visayas	53	7.9
Unknown	6	0.9
No. of isolates per individual**		
1	653	97.3
2	8	1.2
3	2	0.3
4	3	0.4
5	1	0.1
6	3	0.4
14	1	0.1

*Based on the Island of collection for sequenced isolates.

**At different time points.

**Table 2:** *Mycobacterium tuberculosis* samples (N = 724).

Characteristic	N	%
Year of collection		
2011	61	8.4
2012	113	15.6
2013	60	8.3
2014	37	5.1
2015	116	16.0
2016	267	36.9
2017–2019	70	9.7
Lineage		
L1	531	73.3
L4	174	24.0
L2	18	2.5
L3	1	0.1
Drug resistance**		
Sensitive	119	16.4
HR-TB	79	10.9
RR-TB	36	5.0
MDR-TB	374	51.7
Pre-XDR	98	13.5
XDR-TB	3	0.4
Other	15	2.1
Individual resistance		
Rifampicin	511	70.6
Isoniazid	554	76.5
Ethambutol	317	43.8
Ethionamide	282	39.0
Pyrazinamide	223	30.8
Streptomycin	288	39.8
Fluoroquinolones	102	14.1
Aminoglycosides	72	9.9
Amikacin	72	9.9
Capreomycin	92	12.7
Kanamycin	76	10.5
Cycloserine	3	0.4
Para-aminosalicylic acid	19	2.6
Bedaquiline	3	0.4

*HR-TB* isoniazid resistant, *MDR-TB* multidrug-resistant, *XDR* extensive-drug-resistant.

*This does not include 8 mixed infection samples (see Table S1).

**Genotypic; isolates included only in one category.

### Drug resistance

**Figure 1:**
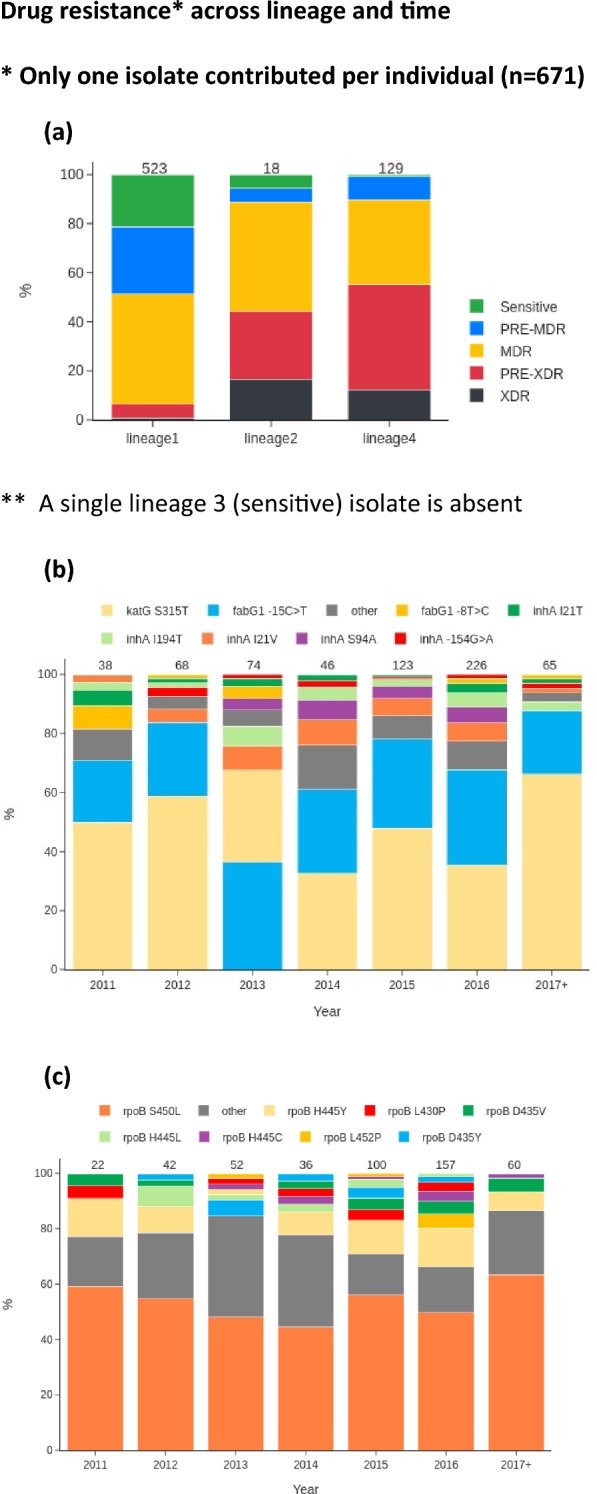
Drug resistance* across lineage and time. *Only one isolate contributed per individual (n = 671). (**a**) Types by lineage. A single lineage 3 (sensitive) isolate is absent. (**b**) Mutations linked to resistance to isoniazid. (**c**) Mutations linked with resistance to rifampicin.

Genotypic drug resistance characterisation was performed using TB-Profiler software (v2) on WGS data from 724 M*. tuberculosis* isolates. The drug-resistance profiles generated span 16 drugs, with resistance prevalent for first-line drugs such as rifampicin (70.6%), isoniazid (76.5%), ethambutol (43.8), and pyrazinamide (30.8%) (Table 2). More than half of all strains (n = 374; 51.7%) were multidrug-resistant tuberculosis (MDR-TB), with high concordance between phenotypic (drug susceptibility testing) and genotypic resistance for rifampicin (93.5%) and isoniazid (91.7%). Furthermore, genotypic resistance to streptomycin (39.8%), fluoroquinolones (14.1%), aminoglycosides (9.9%), and ethionamide (39.0%) was detected. Pre-extensively drug-resistant tuberculosis (pre-XDR-TB), defined as MDR-TB with additional resistance to any fluoroquinolone, was also found in 98 (13.5%) isolates. Similar proportions of isolates with MDR-TB were observed across all lineages except L3 (Fig. 1).

The most common mutations underlying MDR-TB were in *katG* (S315T; 321/554) for isoniazid, *fabG1* promoter region (− 15C>T; 222/554) for isoniazid, and *rpoB* (D435G/F/V/E, 51/511; S450L/F/W, 303/511) for rifampicin (Table 3). Moreover, compensatory mutations were also identified linked to isoniazid (*ahpC* − 48G>A, n = 4; − 52C>T, 3; − 54C>T, 2;  − 74G>A, 1; − 81C>T, 4; − 51G>A, 4; − 52C>A, 3) and rifampicin (*rpoC* D485N, n = 2; I491T, 1; N698K, 8; N698S, 5; L516P, 6; F452L, 1; V483A, 13; V483G, 11). Other common mutations included *embB* (G406A/S/D, 19/343; M306I/L/V, 88/343; Q497R/P, 6/343) linked to ethambutol and *gyrA* (A90V, 32/103; S91P, 3/103; D94G/A/H/Y/N, 65/103) linked to fluoroquinolones. Mutations associated with para-aminosalicylic acid (PAS) resistance were identified in 21 isolates *folC* E40G (n = 18), *folC* R49P (n = 2), and *thyA* R126Q (n = 1). Several resistance mutations, rare in a global dataset (“Global50k”; n = 50,722) (< 1%) were found, including those for isoniazid (*inhA* I21V, I21T), pyrazinamide (*pncA* 316_317dupTT, H57P), and streptomycin (*rrs* 514A>T). Most established resistance mutations were present from 2011 (e.g., *katG* S315T, *rpoB* S450L), while others emerged in later years (e.g., *inhA* − 154G>A, *rpoB* H445T in 2012) (Fig. 1; Fig. S1). Interestingly, some isolates with known resistance mutations had a sensitive phenotype, often due to reported “disputed” mutations other than the canonical *rpoB* S450L^10^. For example, only five (38.5%) of 13 isolates with the *rpoB* L430P mutation had a rifampicin-resistant phenotype. In total, seven known rifampicin resistance mutations (across 38 isolates) had resistant proportions above 50%, but mutation frequencies were uncommon (n < 14) compared to *rpoB* S450L (n = 266/671; 39.6%), H445Y (n = 57/671; 8.5%), and D435V (19/671; 2.8%).

**Table 3:** Common mutations linked to drug resistance.

Drug resistance	Gene name	Change	Our study (n = 724) N (%)	Our study one isolate per individual (n = 671) N (%)	Global 50 k %*
Isoniazid	*katG*	S315T	333 (46.0)	291 (43.4)	61.2
Rifampicin	*rpoB*	S450L	297 (41.0)	266 (39.6)	57.4
Isoniazid	*fabG1*	− 15C>T	236 (32.6)	203 (30.3)	17.9
Ethambutol	*embB*	M306V	121 (16.7)	94 (14.0)	29.2
Streptomycin	*rrs*	514A>C	102 (14.1)	83 (12.4)	8.4
Ethambutol	*embB*	M306I	82 (11.3)	68 (10.1)	22.4
Kanamycin	*rrs*	1401A>G	72 (9.9)	53 (7.9)	55.8
Pyrazinamide	*pncA*	L172P	62 (8.6)	46 (6.9)	0.6
Rifampicin	*rpoB*	H445Y	57 (7.9)	57 (8.5)	3.7
Streptomycin	*rpsL*	K43R	41 (5.7)	39 (5.8)	45.8
Isoniazid	*inhA*	I21V	41 (5.7)	40 (6.0)	0.5
Ofloxacin	*gyrA*	A90V	32 (4.4)	22 (3.3)	25.6
Isoniazid	*inhA*	I194T	29 (4.0)	25 (3.7)	1.3
Ofloxacin	*gyrA*	D94G	28 (3.9)	22 (3.3)	35.0
Ethambutol	*embB*	G406S	28 (3.9)	12 (1.8)	1.9
Rifampicin	*rpoB*	D435V	25 (3.5)	19 (2.8)	7.0
Isoniazid	*inhA*	S94A	24 (3.3)	24 (3.6)	1.4
Ethambutol	*embB*	G406D	24 (3.3)	20 (3.0)	3.0
Streptomycin	*gid*	102delG	22 (3.0)	22 (3.3)	4.2
Rifampicin	*rpoB*	H445D	22 (3.0)	9 (1.3)	3.2
Streptomycin	*rrs*	514A>T	20 (2.8)	8 (1.2)	0.3
Streptomycin	*rpsL*	K88R	20 (2.8)	20 (3.0)	8.7
Rifampicin	*rpoB*	Q432K	16 (2.2)	3 (0.4)	0.2
Pyrazinamide	*pncA*	316_317dupTT	16 (2.2)	3 (0.4)	0
PAS	*folC*	E40G	16 (2.2)	3 (0.4)	0
Streptomycin	*gid*	351delG	15 (2.1)	15 (2.2)	2.0
Streptomycin	*gid*	48delT	15 (2.1)	13 (1.9)	0.2
Isoniazid	*inhA*	I21T	15 (2.1)	15 (2.2)	0.8
Ofloxacin	*gyrA*	D94Y	15 (2.1)	2 (0.3)	3.9
Pyrazinamide	*pncA*	H57P	15 (2.1)	4 (0.6)	0.1
Rifampicin	*rpoB*	L430P	13 (1.8)	13 (1.9)	2.0
Isoniazid	*fabG1*	− 8T>C	13 (1.8)	13 (1.9)	1.6
Rifampicin	*rpoB*	D435Y	13 (1.8)	13(1.9)	2.7
Ethambutol	*embB*	D1024N	12 (1.7)	12 (1.8)	2.1
Streptomycin	*rrs*	517C>T	12 (1.7)	12 (1.8)	4.8
Capreomycin	*tlyA*	269dupG	12 (1.7)	1 (0.1)	0
Ethambutol	*embB*	M306L	11 (1.5)	11 (1.6)	1.4
Rifampicin	*rpoB*	H445L	11 (1.5)	11 (1.6)	1.1

*Frequency in isolates with associated drug resistance in the Global50k database (n = 50,722); PAS Para-aminosalicylic acid.

### Putative novel drug resistance genes

Although there was a high concordance between phenotypic resistance and genotypic predictions (accuracy: rifampicin: 93.5%, isoniazid: 91.7%), some isolates presented a resistant phenotype with no known resistance mutation. These isolates were further analysed to identify potentially novel mutations that could explain resistance. For isoniazid, three mutations were identified across five samples, one in *inhA* (I21M) and two in *katG* (K143E, D419Y) (Table S3). Moreover, the characterization of rare and unknown-association mutations in candidate genes and phenotypic testing of selected strains led to the detection of potential novel resistance mutations for ethambutol (11 mutations across *embA/B/C*), pyrazinamide (two mutations in *pncA*), streptomycin (27 mutations in *gid* and one in *rrs*), fluoroquinolones (one in *gyrA*) and capreomycin (two in *tlyA*). While some of the isolates with these mutations were classified as sensitive according to phenotypic methods, the majority exhibited a resistant phenotype. Additionally, four frameshift mutations across three samples were identified in *mmpR5* (*Rv0678*), which is strongly associated with bedaquiline resistance. While phenotype testing was not available for bedaquiline, frameshift mutations are widely accepted to cause resistance. Additionally, these mutations were acquired on an MDR-TB and fluoroquinolone background, making them XDR-TB.

### Phylogenetic and clustering analysis

**Figure 2:**
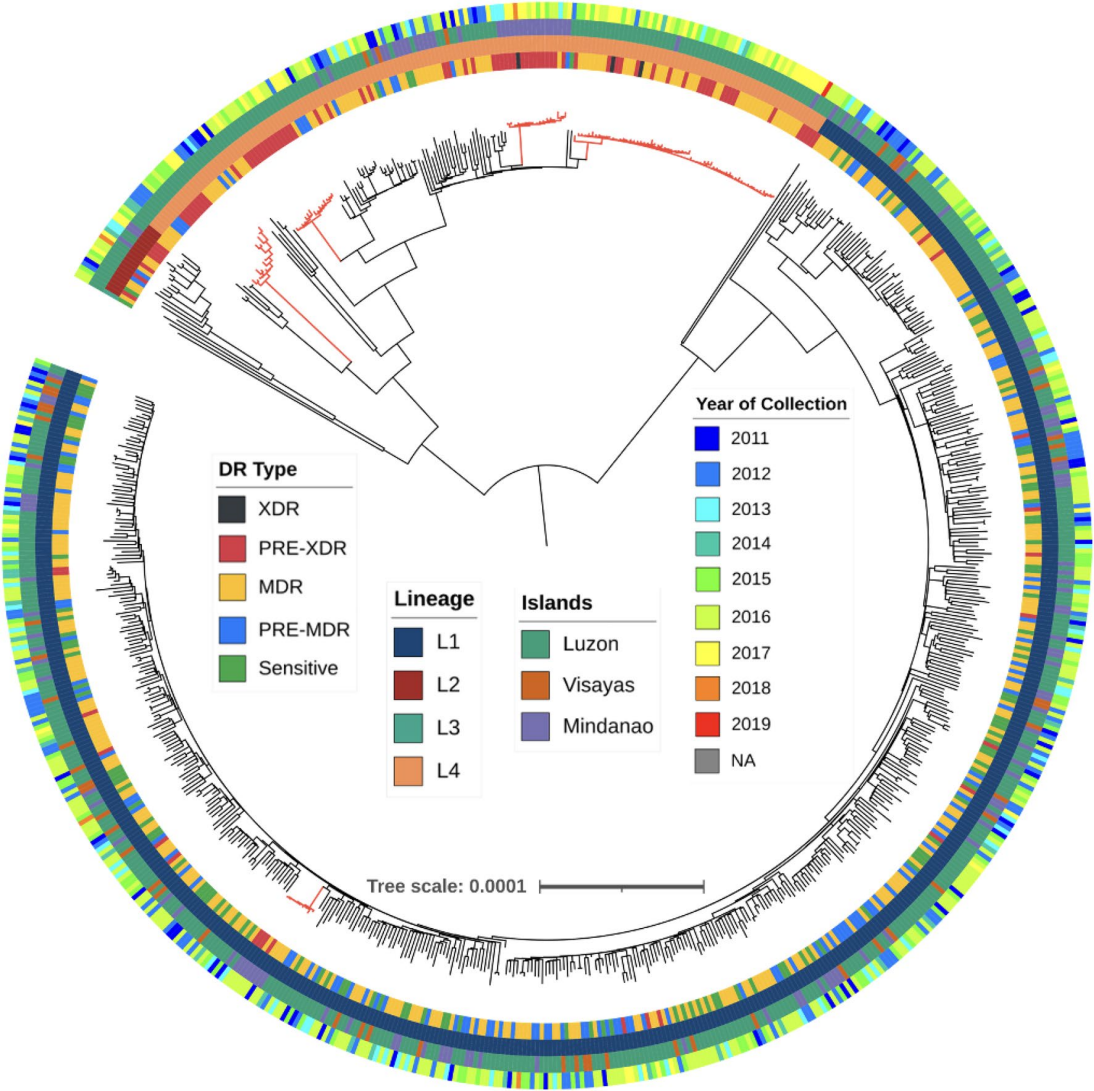
Phylogenetic tree of the 724 M*. tuberculosis* study isolates constructed using 34,260 SNPs. The colour scheme label on the phylogenetic tree from the innermost to outermost ring indicates: Drug Resistance (DR) type, Lineage, Islands, and Year of Collection. The 5 red-coloured clades of highly similar isolates are explored in Fig. 3.

The study detected 34,260 SNPs across all 724 isolates examined, of which 73.8% were unique to a single sample. Phylogenetic tree construction using all SNPs confirmed the expected grouping of the isolates based on their evolutionary lineage. Clades with similar drug-resistance profiles were found, including large clusters of MDR and pre-XDR isolates in L4 (Fig. 2) spanning up to eight years. Prison-sourced isolates (n = 71) from 18 inmates across different time points (range: 2 to 14 isolates per person; from 2013 to 2019 with a median time span of 2 years) explained some of this clustering (Table S4). The prison-sourced isolates were mostly L4 (55/71), predominantly L4.3.4.1 and L4.3.4.2 strains, with some additional representation from L1 (16/71), largely consisting of the Manila strain (L1.2.1.2.1). Using all serially sampled isolates without mixed infections (n = 69), the estimated crude mutation rate was 0.66 SNPs per isolate per year (Fig. S2). To control for potential cryptic reinfections, we excluded isolates with more than one SNP difference and a time interval of less than one year (n = 60), resulting in an estimate of 0.34. An analysis of L4 isolates (48/60) led to a crude rate of 0.35 SNPs per isolate per year. The largest sublineage in our dataset was L1.2.1.2.1 (Manila family), which has been predominantly observed in isolates collected from the Philippines^3^. To characterise the molecular clock rate in this sublineage, a time-based phylogenetic tree was reconstructed using BEAST2 software with parameters similar to those described elsewhere^11^. It revealed a clock rate of 0.63 (95% highest posterior density (HPD): 0.17–1.11) mutations per genome per year.

The distribution of pairwise SNP differences across the 671 isolates (one per individual) (median 368; range: 1–2217 SNPs) was multimodal, with modes representing differences within and between lineages (Fig. S3). A genetic distance threshold of 12 SNPs for defining potential transmission was established by evaluating a range of SNP cut-offs (0 to 30) (Fig. S3, Table S5). This cut-off resulted in 32 clusters containing a total of 120 isolates (Table S5), with a maximum of 45 samples in a single cluster (Fig. S4).

**Figure 3:**
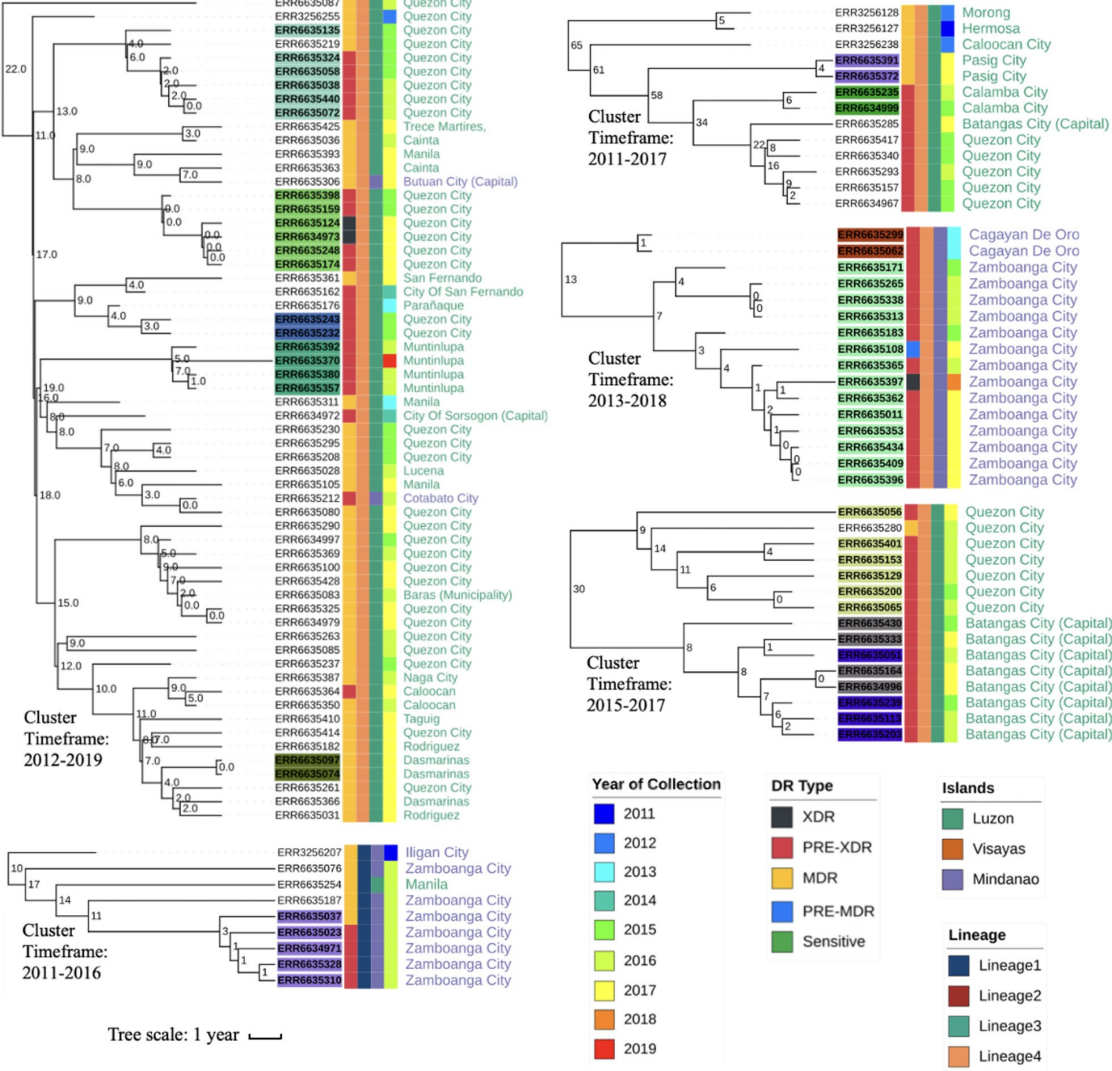
Clustered isolates. Major clusters from Fig. 2, and includes individuals with > 1 sample. Strip colour scheme from left to right: Drug resistance (DR) type, Lineage, Islands, and year of collection. Sample IDs that are highlighted with colours indicate isolates from the same patients. The numbers on the nodes of the tree show the maximum SNP distance between samples in the bifurcating branches. The timeframes of clusters are indicated (earliest and latest years).

An analysis of the pairwise most recent common ancestor across the clusters led to median and mean values of 9 and 8 years, respectively, ranging from 0.6 to 16 years. The 120 isolates in clusters were found in all three islands (Luzon, 89; Visayas, 6; and Mindanao, 25) and were predominantly MDR-TB and pre-XDR (89/120). Logistic regression analysis revealed a greater risk of transmissibility (compared to L1) for L4 (odds ratio (OR) 12.87) and L2 strains (OR 4.02), as well as more advanced drug-resistance (OR 2.16) (all P < 0.001) (Table S6). There was some suggestive evidence of increased transmissibility for isolates from Mindanao compared to those from Luzon (OR 2.18, P = 0.015) (Table S6). A positive association between overall SNP distance and geographical distance was observed (ANOVA F = 346.2, P < 0.001) (Fig. S5). The largest cluster (n = 45) spans 19 cities, seven regions, and two islands, with a high proportion in Luzon, and contains MDR-TB (n = 36) and pre-XDR (n = 9) strains (Fig. S4; Fig. 3). Time-based Bayesian phylogenetic trees of the five largest clusters (from the analysis of 724 isolates) were generated using BEAST2 software^12^, revealing clusters of isolates collected from the same island and geographic units (Fig. 3). Interestingly, the clusters included serial samples from the same patients, which were found to have different levels of drug resistance at different periods while interspaced with the samples from unique sample hosts, suggesting potential direct transmission events between individuals. Additionally, the microevolution of drug resistance was observed within and between hosts, with the progression of MDR-TB to pre-XDR and then to XDR-TB.

A genome-wide association study (GWAS) approach (n = 671) was applied to identify genetic loci associated with transmissibility. This identified signals in the following genes: *Rv0425c, rrs, Rv2828A, Rv3198c, Rv0766c,* and *Rv0825c* genes (Table S7) (all ORs > 3; P < 0.0001). The detected *rrs* mutation 514A>C is associated with streptomycin resistance, while 1401A>G is linked to resistance against kanamycin, capreomycin, and amikacin, according to GWAS analysis. The *rrs* gene encodes 16S ribosomal RNA, which is involved in metabolism and xenobiotic detoxification^13^. The *rrs* 1401A>G is present in three clusters, while *rrs* 514A>C is found in two clusters in L4. The *Rv0425c* (mutation M689V) gene is a metal cation transporting P-type ATPase (CtpH), suggesting a role in membrane maintenance and ion transport^14^. The *Rv2828A* R89W mutation has been previously linked to TB survival and virulence^15^, and it was found in all isolates in the largest cluster, which consists exclusively of L4 isolates. The *Rv0825c* gene is responsible for fatty acid metabolism^16^, and the Gln178* mutation was found in two L4 clusters. The *Rv3198c* gene (D420V mutation) is linked to a probable ATP-dependent DNA helicase II known as UvrD2, which maintains bacterial genome integrity^17^. The *Rv0766c* (G337C mutation) gene is linked to cytochrome P450, Cyp123, which is involved in cellular metabolism and xenobiotic detoxification^18^. *Rv3198c* D420V and *Rv0766c* G337C mutations are only found in the large L4 cluster. Lastly, the *Rv3092c* gene (P250L mutation) may influence the function of a conserved integral membrane protein^19^.

## Discussion

WGS is increasingly being used to diagnose and track TB infections, and the Philippines, a high-burden TB country, has growing investments in such genomic technologies. WGS of isolates from previous Philippine TB prevalence surveys revealed circulating L1 and L4 strains, including MDR-TB and XDR-TB forms in clustered sequences. While “ancient” L1 Manila strains are considered the most prevalent circulating strains, evidence of “modern” L4 drug-resistant strains within prisons was found, consistent with a previous study (n = 25) that characterised strain types using spoligotyping and MIRU-VNTR typing^20^. Although our *M. tuberculosis* isolates were pseudo-randomly selected and considered a convenience sample of mostly drug-resistant isolates, which may not reflect circulating allele frequencies, much-needed insights into resistance mutations and related transmission events were gained. Five large clusters of highly similar isolates were identified on the islands of Luzon and Mindanao, and the underlying isolates clustered by geography through the Bayesian dated phylogenetic reconstruction. Using a GWAS approach, several loci (e.g., Rv0425c, *rrs*, Rv2828A, Rv3198c, Rv0766c, and Rv0825c) associated with the “transmissibility” phenotype were identified, primarily in L4 clusters, which could be linked to increased *M. tuberculosis* fitness and transmission. Whilst the Rv2828A locus has been previously linked to TB survival and virulence^15^, the relevance of the identified genes on transmission should be investigated through prospective collections, analysis of other populations, and experiments on gene function.

Using serially sampled isolates, we inferred a crude mutation rate of 0.41 SNPs per year. It has been shown that different lineages of *M. tuberculosis* evolve at different rates^11^. Serial isolates can be from re-infections of independent but closely related strains in the patients rather than evolution of the bacteria within a single patient, both leading to inaccurate calculations^11^. By excluding isolates with > 1 SNP and less than one year difference, the estimate was 0.66 SNPs per isolate per year, whilst an analysis of L4 isolates revealed a rate of 0.35. Using BEAST2 software, the molecular clock rate of the predominant L1.2.1.2.1 Manila family was estimated at 0.63 (95% HPD: 0.17–1.11) mutations per genome per year. Although, the clock rates are consistent with estimates of other lineages, the Manila family could be faster than L4 and may contribute to the observed relatively higher transmission rates of the L1 strain type^4,11^. A more comprehensive analysis involving other L1 strain types is required to confirm any molecular clock differences in the Manila family. We used the most recent isolate to avoid bias from using serial isolates in some population-based analyses, including when estimating mutation frequencies.

WGS data also revealed the presence of XDR-TB strains with genotypic resistance to isoniazid, rifampicin, fluoroquinolones and bedaquiline for the first time in the Philippines. Three isolates from two hosts had frameshift mutations in *mmpR5* (144dupC, 198dupG, and 135delG) strongly associated with bedaquiline resistance. Interestingly, all three mutations were not found at fixed frequencies in the population, suggesting that they may have been collected while the genetic heterogeneity was still present in the host bacterial population. Remarkably, all three mutations were found across two samples from the same host in the same year, with one sample containing 144dupC at 17% within-sample abundance, and the other isolate containing 198dupG at 60% and 135delG at 32% abundance. This observation indicates that three independent acquisitions of bedaquiline resistance mutations occurred in the same host. The other host had a sample containing 144dupC at 55%. Both hosts were in prison at the time of collection. This finding suggests that XDR-TB is developing in Philippine prisons, with the potential to spread to the community. Indeed, Bayesian phylogenetic reconstruction indicated samples from prisons clustering closely and sometimes interspaced with those collected from community settings. Additionally, two of the eight mixed-strain infections identified were sourced from prisoners. These observations suggest that prisons are a potential reservoir of highly resistant and transmissible TB. A previous study of TB in Filipino prisons (n = 25) used genotyping methods to identify two potential clusters and 23 genotypes^20^, but WGS provides a much higher resolution of transmission^4^. Public health measures for adequately managing such cases are imperative to prevent onward transmission.

Although phenotypic drug susceptibility testing (DST) and genotypic predictions for MDR-TB were highly concordant (> 90%), our analysis of discordant cases revealed three putative novel markers for isoniazid (*inhA* I21M, *katG* K143E and D419Y). The three markers are currently classified as having uncertain significance. However, the findings from this study bolster the confidence in a potential association between these three SNPs and their corresponding drugs^21^. We also identified a number of potentially novel resistance mutations in candidate genes for other drugs, including streptomycin (n = 27 novel mutations), ethambutol (n = 11), pyrazinamide (n = 2), capreomycin (n = 2), and fluoroquinolones (n = 1). These rare mutations were supported by phenotypic DST data and the large Global50k database (n = 50,722) of strains, ruling out phylogenetic-specific mutations. Interestingly, rifampicin DST data showed that samples with established resistance mutations in *rpoB* other than S450L had much higher odds of presenting a sensitive phenotype. This result is consistent with previous reports^22^, and has been linked to slower growth on Mycobacteria growth indicator tube (MGIT) assays. This observation implies that individuals could be prescribed suboptimal regimens with rifampicin that are not effective, highlighting the strengths of using NGS to identify these cases.

Overall, this study confirms the advantages of using whole genome approaches to characterise drug resistance profiles and transmission patterns. With the advent of affordable and accessible rapid WGS or targeted amplicon sequencing diagnostics, the generated sequences and identified mutations provide a baseline set of comparative data for future applications. These include integrating machine learning algorithms and databases with informative drug resistance markers. A deeper understanding of transmission dynamics across time and geography through routine surveillance will help prioritise infection control resources and activities. Surveillance programs should also record and share novel drug resistance SNPs to coordinate a global response. Ultimately, these insights will inform clinical and public health decision-making, contributing to significant reductions in the burden of TB.

## Materials and methods

### DNA extraction and sequencing

A total of 475 M*. tuberculosis* short-term cultured isolates from sputum samples collected by the Research Institute for Tropical Medicine (RITM) in the Philippines between 2012 and 2019 were pseudo-randomly selected for the study. Informed consent was obtained from all subjects and/or their legal guardian(s). This study was given authorisation by the Institutional Review Board of the RTIM (ID No. RITM-IRB 2017–05). Drug susceptibility testing was performed as part of routine TB culture and phenotypic assessments in BSL3 laboratories at the RITM for rifampicin and isoniazid, and additionally for some isolates for ethambutol, streptomycin, amikacin, kanamycin, capreomycin and levofloxacin drugs (see protocols elsewhere^3^). Total genomic DNA was extracted using a phenol–chloroform extraction procedure. DNA extract concentrations and quality were measured using a Qubit fluorometer (Life Technologies Holdings Pte Ltd, Singapore) and were visualised with a 1% agarose gel. Library preparation of the DNA samples was performed using a QIAseq FX DNA library kit, following the manufacturer’s protocol. We quantified the libraries using dsDNA Qubit Assay, while the libraries’ sizes were measured using Agilent Tapestation 2200 DNA 1000 assay kit. At the Philippine Genome Center (Manila), we normalized to 4 nM, pooled and sequenced all H37Rv libraries using Illumina Novaseq 6000 (2 × 151 base pair reads). WGS data was also available from previous studies (n = 257), including DRS2 (Second National TB Drug Resistance Survey in 2012) (n = 166; years 2011–2012)^3^ and NTPS (National Tuberculosis Control Program Survey) (n = 91, year 2016)^23^. The combined dataset consisted of 732 isolates with WGS data. All raw sequencing data is available (see Supplementary Data 1 for a list of accession numbers). All methods were performed in accordance with the relevant guidelines and regulations.

### Bioinformatic and statistical analyses

Sequence reads were inspected using fastQC (www.bioinformatics.babraham.ac.uk/projects/fastqc/) as a primary data quality assessment. The reads were trimmed using trimmomatic^24^ (v0.38; LEADING:3 TRAILING:3 SLIDINGWINDOW:4:20 MINLEN:36) to remove low-quality sequences, and then mapped against the H37Rv reference genome (AL123456) using BWA-mem^25^ (v0.7.17). SNPs were called using the BCF/VCF tool suite (v1.8)^26^ in regions with at least 10 reads. SNPs were removed from non-unique regions of the genome (e.g., *ppe* genes). Full-length consensus genomes were created by inserting SNPs for each sequence into the H37Rv reference using the bcftools consensus tool. Consensus genomes were masked at low-coverage positions (< tenfold depth) and within genomic regions that are difficult to characterise with short-read sequencing (e.g. *pe/ppe* genes). Consensus genomes were concatenated and used as input to iq-tree (v2.2.2.7)^27^ to reconstruct the phylogeny. BEAST2 software^12^ was used to construct an MCMC phylogenetic trees, with parameter settings that calibrated the time scale using alignments without invariant positions^28^. Lastly, ITOL software was used to visualise the trees^29^. Drug resistance profiles and lineages were predicted *in-silico* using TB-Profiler software (v2.0). Variant annotations were labelled using SnpEff software^30^.

Mixed infections were found using Gaussian mixture modelling^30,31^ of SNP allele coverage data, leading to eight samples being removed and full analysis being performed on 724 isolates. The distribution of SNP genotype differences between isolates (pairwise) was used to determine a transmission cut-off (of 12), which was sufficiently stringent to avoid expected (sub-)lineage differences (Fig. S3). The association of the presence in clustered isolates (yes/no) with lineages and drug resistance was explored using logistic regression models, leading to odds ratios. To determine SNPs linked to potential transmissibility, GWAS was performed using a logistic regression model in Plink2 software^32^, which adjusted for lineage and drug resistance. This approach has been applied previously^33^. Mutation frequencies for SNPs of interest were compared to those from an *M. tuberculosis* database (“Global50k”; n = 50,722^34^), which covers all lineages across > 100 countries. We used the current definitions of drug-resistant TB: we defined MDR-TB as TB resistant to isoniazid and rifampicin, pre-XDR TB as MDR-TB with resistance to any fluoroquinolone, and XDR-TB as MDR-TB with additional resistance to any fluoroquinolone and another WHO group A drug (bedaquiline or linezolid)^35^.

### Supplementary Information

Supplementary Information 1.

Supplementary Information 2.

## Data Availability

Previously published and newly generated data can be found on the ENA using the Run accession codes in Supplementary Data 1. The newly generated data can be found under the ENA study accession number ERP114520.
