# Supplementary material for: Whole genome sequencing analysis of Mycobacterium tuberculosis reveals circulating strain types and drug-resistance mutations in the Philippines
Source: Sci Rep. 2024 Aug 23;14:19602. doi: 10.1038/s41598-024-70471-x (PMC11344074; doi:10.1038/s41598-024-70471-x)
Supplement: Supplementary file 2 — Supplementary Information 2. [file 41598_2024_70471_MOESM2_ESM.docx]

# **SUPPLEMENTARY INFORMATION**

**S1 Figure**

**Presence of drug resistance mutations**

1. **Ethambutol**


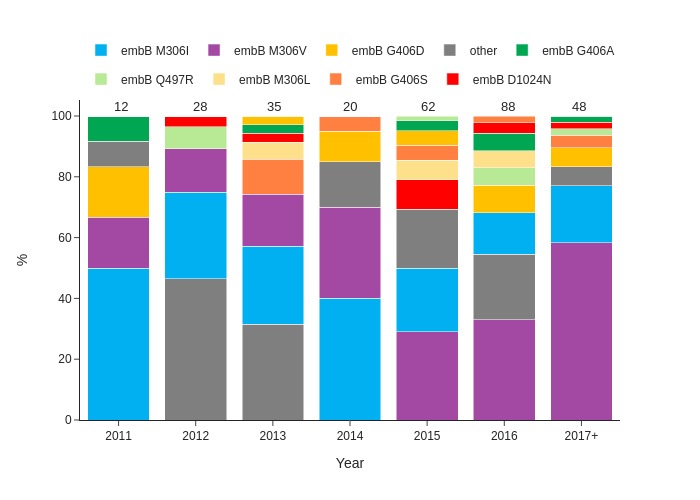


1. **Streptomycin**


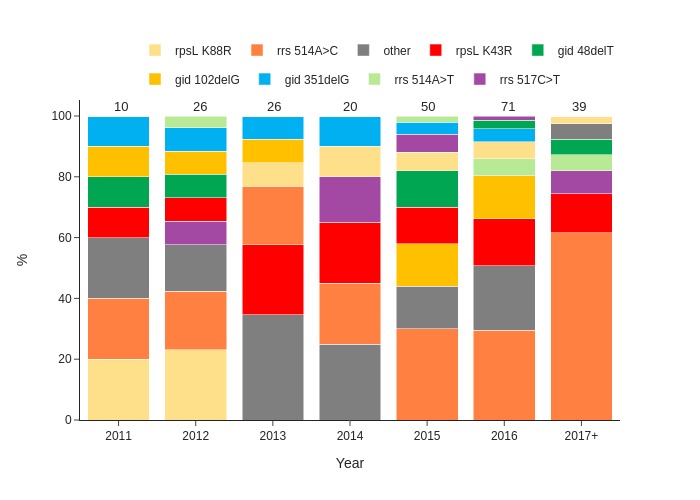


**S2 Figure**

**Mutation rate estimation based on within-patient isolates (n=60)
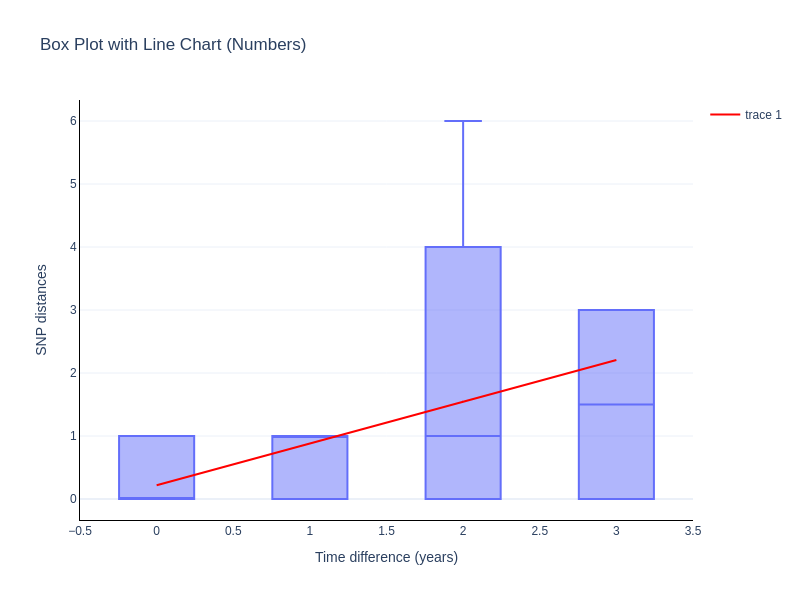
**

The slope of the red line (mutation rate per isolate per year) is 0.663

**S3 Figure**

**Distribution of SNP distances**

1. **All samples (n=724)**

**
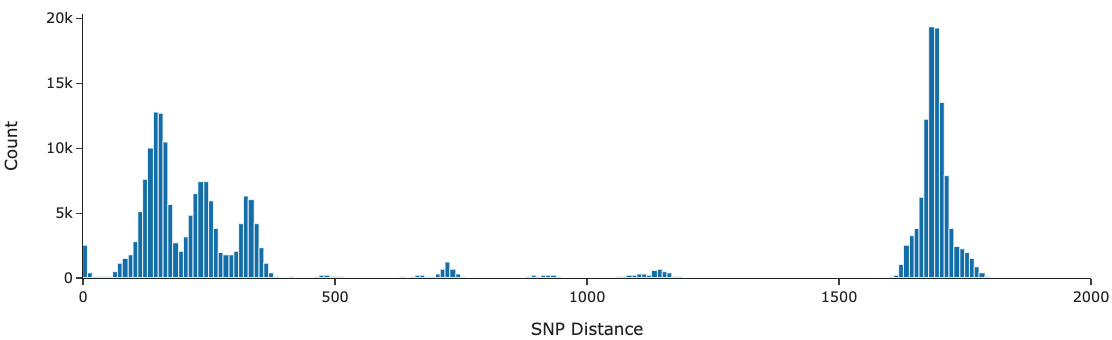
**

1. **Differences of at most 30 SNPs**


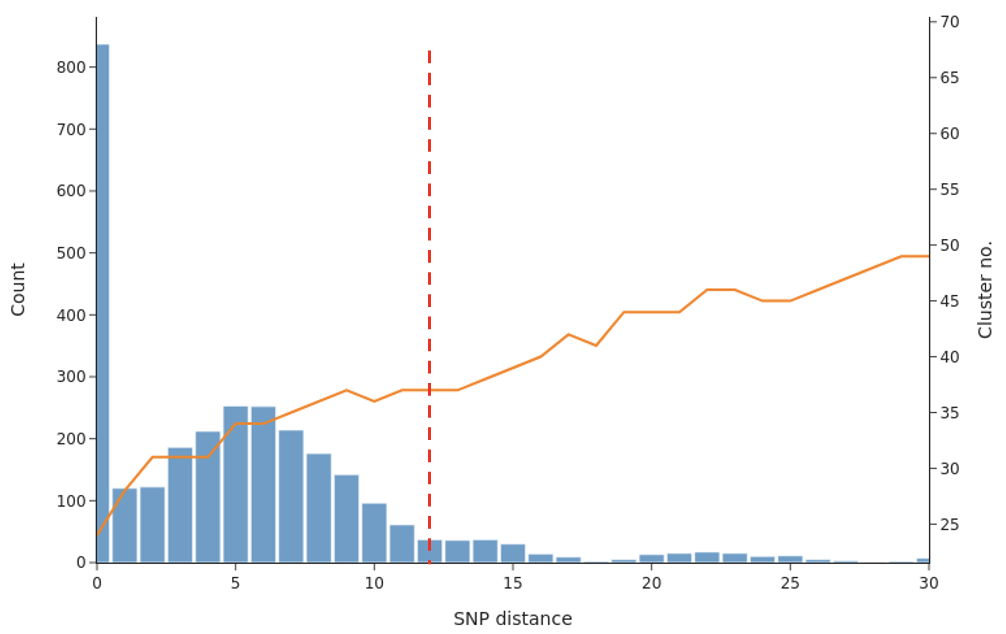


The orange curve shows the number of clusters formed at given SNP distance cut-offs. A cut-off of 12 captured most SNP-distances in the first peak distribution in the multi-modal mixture (red dashed line). The segment from 0 to 12 SNPs covers closely related clustered isolates, and excludes clusters of non-similar isolates within the same and different lineages (other peaks in **(a)**).

**S4 Figure**

**Four largest clusters (based on at most 12 SNPs difference*)
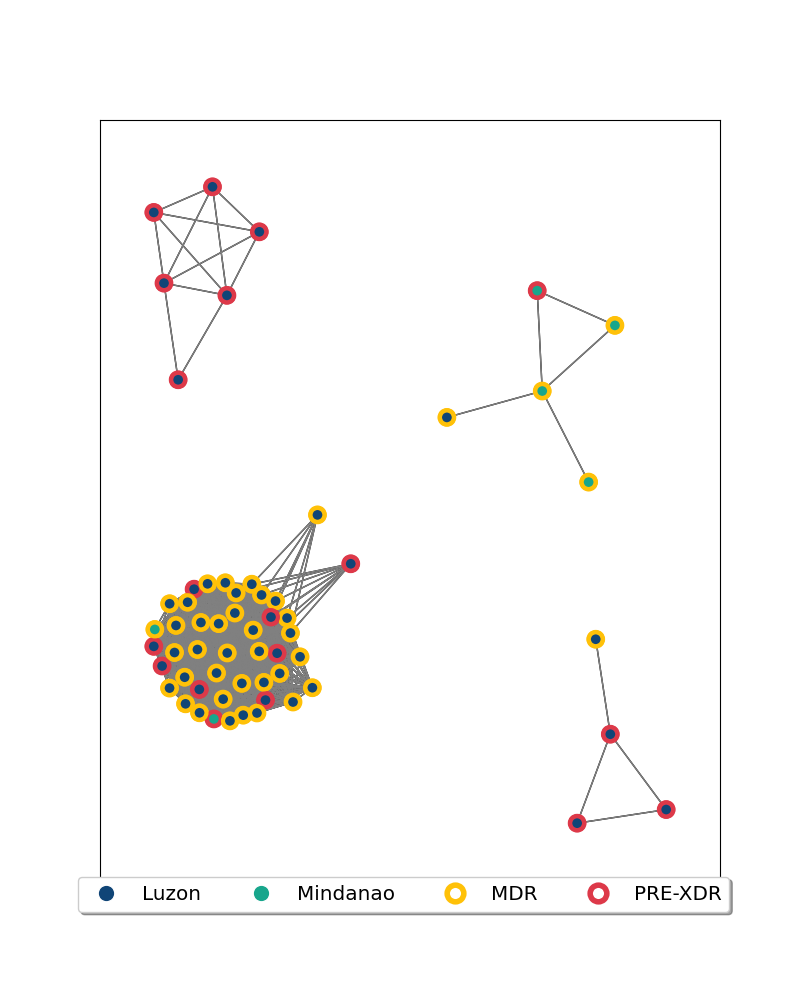
**

* Clusters containing <4 isolates not shown. All clusters consist of isolates from Lineage 4, except for the upper right cluster, made up of isolates belonging to Lineage 1.

**S5 Figure**

**The relationship between SNP and geographical distance**


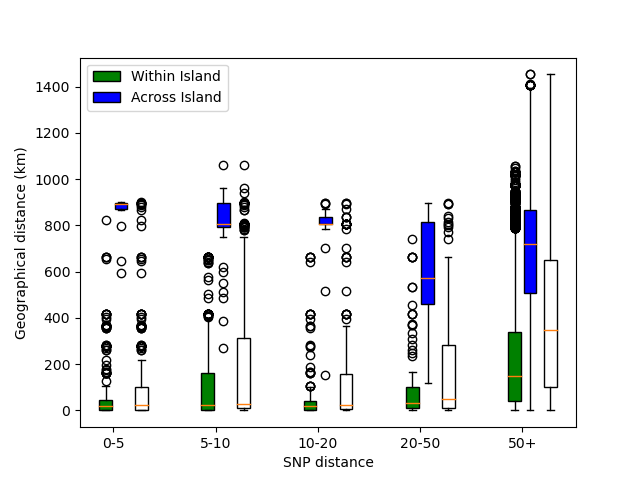


Boxplots show the geographical distance distributions for isolates sourced from patients within (green) and between (blue) islands, and overall (white), at different SNP distance ranges

**S1 Table**

**Mixed infections (n=8) removed from analyses**

| **ID** | **Lineages*** | **Drug resistance profile** | **Mutations** |
| --- | --- | --- | --- |
| ERR6635399 | 1.2.1.2.1 (0.77)  4.3.4 (0.23) | ETH, EMB, RIF, INH | *rpoB* S450L, *rpoB* P483L, *fabG1* -15C>T, *inhA* -154G>A; *embA* D4N; *embB* S297A |
| ERR6635423 | 1.2.1.2.1 (0.80) 2.2.1 (0.20) | CAP, ETH, EMB, RIF, INH, STR | *rpoB* S450L; *katG* S315T, *rpoB* H445D, *rpsL* L43R, *tlyA* L118P, *embB* M306V, *ethA* 1051delG |
| ERR6635381 | 4.3.4.1 (0.38)  4.5 (0.32)  1.2.1.2.1 (0.30) | RIF, INH, PZA, STR, KAN, CAP, AG, AMK, EMB, ETH, | *rpoB* S450L, *katG* S315T, *pncA* H57P, *rpsL* L43R, *rrs* 514A>C, *rrs* 1401A>G, *pncA* L172P, *embB* M306V |
| ERR6635420 | 4.3.4.1  (0.51)  1.2.1.2.1  (0.49) | RIF, INH, EMB, PZA, STR, AMK, CAP, ETH, KAN | *rpoB* H445T, *rpoB* S450L, *katG* S315T, *fabG1* -15C>T, *pncA* L172P, *embB* M306V, *embB* D1024N, *rpsL* L43R, *rrs* 514A>C, *rrs* 1401A>G |
| ERR6635384 | 4.3.4.1  (0.54)  1.2.1.2.1  (0.45) | AG, PAS, AMK, CAP, ETH, EMB, RIF, INH, KAN, PZA, STR | *katG* S315T, *fabG1* -15C>T |
| ERR6635386 | 4.3.4.1 (0.59)  1.2.1.2.1 (0.33) | RIF, INH, FQ, STR, ETH, PZA, PAS, EMB | *rpoB* S450L, *katG* S315T, *gyrA* A90V, *rpoB* H445T, *rpsL* K43A, *rrs* 514A>C, *rrs* 1401A>G, *fabG1* -15C>T, *pncA* L172P, *folC* E40G, *embB* M306V, |
| ERR6635343 | 4.3.4.2 (0.63)  1.2.1.2.1 (0.37) | RIF, INH | *rpoB* D435Y, *katG* S315T |
| ERR6635323 | 1.2.1.2.1 (0.94)  4 (0.05) | RIF, INH, PZA, EMB, ETH, STR | *rpoB* S450L, *katG* S315T, *pncA* D63A, *embB* M306I, *gid* A118fs, *fabG1* -15C>T, *inhA* S94A, *embB* Y319S, |

* Lineage number (proportion); AG – Aminoglycosides, AMK – Amikacin, CAP – Capreomycin, EMB – Ethambutol, ETH – Ethionamide, FQ – Fluoroquinolones, INH – Isoniazid, KAN – Kanamycin, PAS - Para-aminosalicylic acid, PZA -Pyrazinamide, RIF – Rifampicin, STR - Streptomycin

**S2 Table**

**Number of phenotypically sensitive and resistant isolates reported with known rifampicin resistance mutations**

| ***rpoB* mutation** | **Frequency** | **No. Resistant** | **% Resistant** |
| --- | --- | --- | --- |
| S450L | 266 | 262 | 98.4 |
| H445Y | 57 | 56 | 98.2 |
| L430P | 13 | 5 | 38.5 |
| H445L | 11 | 9 | 81.8 |
| H445C | 10 | 9 | 90.0 |
| L452P | 10 | 7 | 70.0 |
| H445N | 4 | 2 | 50.0 |
| H445S | 4 | 1 | 25.0 |
| S493L | 2 | 1 | 50.0 |
| I491F | 1 | 0 | 0.0 |
| P483L | 1 | 0 | 0.0 |

**S3 Table**

**High confidence unreported SNPs potentially linked to drug resistance**

| **Drug** | **Gene** | **SNP** | **Phenotypic* Sensitive** | **Phenotypic* Resistant** |
| --- | --- | --- | --- | --- |
| Isoniazid | *inhA* | I21M | 0 | 1 |
|  | *katG* | K143E | 0 | 3 |
|  |  | D419Y | 0 | 1 |
| Ethambutol | *embA* | -8C>T | 1 | 1 |
|  |  | -43G>C | 2 | 3 |
|  |  | -28delT | 0 | 1 |
|  |  | P61H | 0 | 1 |
|  | *embB* | G603E | 0 | 1 |
|  |  | G603R | 0 | 1 |
|  |  | L1036V | 1 | 1 |
|  |  | C361Y | 0 | 1 |
|  |  | A249V | 1 | 1 |
|  |  | N318D | 1 | 1 |
|  | *embC* | A664V | 0 | 1 |
| Pyrazinamide | *pncA* | 287_289dup | 0 | 1 |
|  |  | T47I | 0 | 7 |
| Streptomycin | *gid* | G157A | 0 | 1 |
|  |  | 545delG | 0 | 1 |
|  |  | R83W | 0 | 1 |
|  |  | W45* | 1 | 2 |
|  |  | 48delT | 5 | 10 |
|  |  | W45R | 0 | 1 |
|  |  | C52W | 0 | 1 |
|  |  | S181* | 0 | 1 |
|  |  | E120K | 0 | 2 |
|  |  | A167D | 0 | 1 |
|  |  | G157R | 0 | 1 |
|  |  | 193delG | 0 | 1 |
|  |  | W45G | 0 | 17 |
|  |  | 36dupC | 1 | 1 |
|  |  | P75S | 2 | 1 |
|  |  | G117V | 0 | 2 |
|  |  | G69S | 2 | 2 |
|  |  | R137L | 0 | 1 |
|  |  | L142F | 0 | 2 |
|  |  | L74S | 0 | 1 |
|  |  | R47W | 0 | 1 |
|  |  | F12V | 1 | 1 |
|  |  | L49P | 1 | 1 |
|  |  | 99_125del | 0 | 1 |
|  |  | F12S | 0 | 4 |
|  |  | 100_125del | 0 | 1 |
|  |  | G13R | 0 | 1 |
| Streptomycin | *rrs* | n.908A>C | 1 | 2 |
| Fluoroquinolones | *gyrA* | S263F | 0 | 3 |
| Capreomycin | *tlyA* | E142* | 0 | 7 |
|  |  | G90A | 0 | 5 |

*Phenotypic DST result for corresponding resistance

**S4 Table**

**Study participants* contributing serial isolates**

| **Patient ID** | **Year of collection** | **Lineage** | **Count** |
| --- | --- | --- | --- |
| 1 | 2015 | 4.3.4.1 | 2 |
| 2 | 2015 | 4.3.4.1 | 2 |
| 2 | 2017 | 4.3.4.1 | 4 |
| 3 | 2013 | 4.3.4.1 | 2 |
| 4 | 2014 | 1.2.1.2.1 | 2 |
| 5 | 2017 | 4.1.1.3 | 2 |
| 6 | 2017 | 4.3.4.1 | 2 |
| 7 | 2015 | 4.3.4.1 | 3 |
| 7 | 2016 | 4.3.4.1 | 3 |
| 8 | 2016 | 1.2.1.2.1 | 2 |
| 9 | 2015 | 4.1.1.3 | 1 |
| 9 | 2016 | 4.1.1.3 | 1 |
| 10 | 2015 | 4.3.4.2 | 1 |
| 10 | 2016 | 4.3.4.2 | 3 |
| 11 | 2015 | 4.3.4.2 | 1 |
| 11 | 2017 | 4.3.4.2 | 3 |
| 12 | 2013 | 4.7 | 1 |
| 12 | 2016 | 1.2.1.2.1 | 1 |
| 13 | 2014 | 1.2.1.2 | 1 |
| 13 | 2016 | 1.2.1.2 | 2 |
| 14 | 2016 | 4.3.4.1 | 3 |
| 14 | 2019 | 4.3.4.1 | 1 |
| 15 | 2017 | 4.3.4.2 | 3 |
| 16 | 2016 | 1.2.1.2.1 | 5 |
| 17 | 2015 | 4.3.4.2 | 1 |
| 17 | 2016 | 4.3.4.2 | 4 |
| 17 | 2017 | 4.3.4.2 | 1 |
| 18 | 2015 | 4.3.4.1 | 2 |
| 18 | 2016 | 4.3.4.1 | 4 |
| 18 | 2017 | 4.3.4.1 | 7 |
| 18 | 2018 | 4.3.4.1 | 1 |

*Not shown, two mixed strain infections that were found in the prison samples and removed from analysis: (i) Patient ID 7 (2016) consisting of lineages 4.3.4.1 and 1.2.1.2.1; (ii) Patient ID 14 (2016) consisting of lineages 4.3.4.1, 4.1.1.3, and 1.2.1.2.1

**S5 Table**

**Clustering by SNP distance**

| **SNP dist.** | **No. Clust.** | **N** | **Max size*** | **L1**  **%** | **L2**  **%** | **L3**  **%** | **L4**  **%** | **MDR**  **%** | **PreMDR**  **%** | **Pre**  **XDR %** | **XDR**  **%** | **Sens.**  **%** |
| --- | --- | --- | --- | --- | --- | --- | --- | --- | --- | --- | --- | --- |
| 0 | 13 | 33 | 7 | 30.3 | 0 | 0 | 69.7 | 36.4 | 27.3 | 18.2 | 12.1 | 6.1 |
| 5 | 26 | 100 | 43 | 28 | 6.0 | 0 | 66 | 42.0 | 30.0 | 14 | 7.0 | 7.0 |
| 10 | 32 | 117 | 44 | 31.6 | 5.1 | 0 | 63.2 | 39.3 | 34.2 | 14.5 | 6.0 | 6.0 |
| **12** | **32** | **120** | **45** | **32.5** | **5.0** | **0** | **62.5** | **39.2** | **35.0** | **14.2** | **5.8** | **5.8** |
| 15 | 34 | 124 | 45 | 33.1 | 4.8 | 0 | 62.1 | 38.7 | 36.3 | 13.7 | 5.6 | 5.6 |
| 20 | 40 | 141 | 46 | 36.2 | 4.3 | 0 | 59.6 | 40.4 | 36.2 | 12.1 | 6.4 | 5.0 |
| 25 | 41 | 147 | 46 | 36.1 | 5.4 | 0 | 58.5 | 40.8 | 36.1 | 11.6 | 6.1 | 5.4 |
| 30 | 46 | 159 | 46 | 37.7 | 6.3 | 0 | 56 | 41.5 | 35.2 | 11.3 | 6.3 | 5.7 |

* Median cluster size for all cut-offs is 2; L# is the lineage; MDR = multidrug resistant; XDR = extensively drug resistant; Sens. = Pan susceptible; a SNP cut-off of 12 captured the majority of SNP-distances in the first Gaussian distribution in the multi-model mixture (**S3 Figure**)

**S6 Table**

**Characteristics linked to transmissibility***

| **Comparison** | **Odds ratio** | **95% CI** | **P-value** |
| --- | --- | --- | --- |
| L2 vs. L1 | 4.01 | (1.33, 12.18) | 0.014 |
| L4 vs. L1 | 12.87 | (7.72, 21.46) | <0.001 |
| Increasing Drug resistance | 2.16 | (1.51, 3.10) | <0.001 |
| Mindanao vs. Luzon | 2.18 | (1.16, 4.08) | <0.001 |
| Visayas vs. Luzon | 2.49 | (0.89, 6.98) | 0.082 |

* *M. tuberculosis* isolates in closely related clusters based on a SNP distance of 12 (n=120) vs. not (n=551); L# = lineage #; CI confidence interval

**S7 Table**

**Mutations associated with transmissibility****

| **Gene** | **Mutation** | **Odds ratio**  ******* | **95% CI** | **-log10p** | **Gene Function** |
| --- | --- | --- | --- | --- | --- |
| *Rv0425c* | M689V | 3.3 | (2.4, 4.4) | 14.7 | Possible metal cation transporting P-type ATPase CtpH |
| *rrs* | 514A>C | 5.1 | (2.8, 9.3) | 6.9 | 16S ribosomal RNA, streptomycin |
|  | 1401A>G | 8.6 | (3.1, 24.1) | 4.4 |  |
| *Rv2828A* | R89W | 7.2 | (3.2, 16.4) | 5.6 | A putative role in TB survival/virulence |
| *Rv3198c* | D420V | 17.8 | (4.3, 73.0) | 4.2 | ATP-dependent DNA helicase II UvrD2 |
| *Rv0766c* | G337C | 12.9 | (3.6, 46.2) | 4.1 | Probable cytochrome P450 123 Cyp123 |
| *Rv0825c* | D178* | 6.7 | (2.6, 17.5) | 4.0 | Essential gene found to play a role in the metabolism of fatty acids |

** *M. tuberculosis* isolates in closely related clusters based on a SNP distance of 12 (n=120) vs. not (n=551); *** adjusted for island, lineage, and drug resistance; CI confidence interval

**S1 Data**

The ENA Run accession identifiers for the samples used can be found in the file *Accession_codes.csv*
